# Direct Visualization of the Highly Polymorphic *RNU2* Locus in Proximity to the *BRCA1* Gene

**DOI:** 10.1371/journal.pone.0076054

**Published:** 2013-10-11

**Authors:** Chloé Tessereau, Monique Buisson, Nastasia Monnet, Marine Imbert, Laure Barjhoux, Caroline Schluth-Bolard, Damien Sanlaville, Emmanuel Conseiller, Maurizio Ceppi, Olga M. Sinilnikova, Sylvie Mazoyer

**Affiliations:** 1 «Genetics of Breast Cancer» team, Cancer Research Centre of Lyon, CNRS UMR5286, Inserm U1052, Université Claude Bernard Lyon 1, Centre Léon Bérard, Lyon, France; 2 Genomic Vision, Bagneux, Paris, France; 3 Service de Génétique, Laboratoire de Cytogénétique Constitutionnelle, Centre de Biologie et de Pathologie Est, Hospices Civils de Lyon and CNRS UMR5292, Inserm U1028, Université Claude Bernard Lyon 1, Equipe TIGER, Lyon, France; 4 Unité Mixte de Génétique Constitutionnelle des Cancers Fréquents, Hospices Civils de Lyon/Centre Léon Bérard, Lyon, France; Florida State University, United States of America

## Abstract

Although the breast cancer susceptibility gene *BRCA1* is one of the most extensively characterized genetic loci, much less is known about its upstream variable number tandem repeat element, the *RNU2* locus. *RNU2* encodes the U2 small nuclear RNA, an essential splicing element, but this locus is missing from the human genome assembly due to the inherent difficulty in the assembly of repetitive sequences. To fill the gap between *RNU2* and *BRCA1*, we have reconstructed the physical map of this region by re-examining genomic clone sequences of public databases, which allowed us to precisely localize the *RNU2* array 124 kb telomeric to *BRCA1*. We measured by performing FISH analyses on combed DNA for the first time the exact number of repeats carried by each of the two alleles in 41 individuals and found a range of 6-82 copies and a level of heterozygosity of 98%. The precise localisation of the *RNU2* locus in the genome reference assembly and the implementation of a new technical tool to study it will make the detailed exploration of this locus possible. This recently neglected macrosatellite could be valuable for evaluating the potential role of structural variations in disease due to its location next to a major cancer susceptibility gene.

## Introduction

Structural variation in the human genome has gained considerable attention in the recent years as it accounts for much of the variation between human genomes and may represent the main genetic basis of phenotypic differences. These variations may also provide an explanation for the missing heritability of complex diseases. Indeed large deletions, duplications, translocations and inversions have potentially great effects, including the changing of gene structure and dosage, altering gene regulation and exposing recessive alleles [1]–[2]. CNVs (Copy Number Variations), the most prevalent type of structural variation in the human genome, refer to DNA segments greater than 1 kb in size that are present at variable copy number [2]–[3]. The assessment of CNV phenotypic and pathologic potency has been made easier recently by the great improvement of CNV maps [4]. According to the high-resolution recent maps, most CNVs in the array-accessible regions of the genome are ancient bi-allelic polymorphisms that are in linkage disequilibrium (LD) with SNPs (Single Nucleotide Polymorphisms). This implies that the contribution of most common CNVs to human phenotypic variation was already detectable in genome-wide association studies (GWAS) as associations to nearby SNPs [5]. However, the question of the implication of multi-allelic CNVs in complex traits remains largely open as most of them cannot be genotyped by array technology, especially macrosatellites, the largest variable number tandem repeats (VNTR) [6]. Some, among which long-published and well documented structural variations, are not even present on the reference genome-assemblies, so their sequence is discarded when alternative genotyping technologies such as next generation sequencing are used [7]. One of such missing CNVs is the *RNU2* locus, which is all the more detrimental that this highly polymorphic macrosatelllite sits next to a major cancer predisposing gene, *BRCA1*.

The *RNU2* gene is transcribed by RNA polymerase II to give the U2 small nuclear RNA (snRNA), an essential component of the spliceosome. In 1984 it was found to lie within a 6.1 kb unit organised as a nearly perfect tandem array of 10 to 20 copies per haploid genome [8]–[9]. It was subsequently localised on chromosome band 17q21q22 [10] to an adenovirus 12-induced metaphase chromosome fragility site [11], in close proximity to the *BRCA1* gene according to FISH (Fluorescent *In Situ* Hybridization), radiation hybrid, physical and genetic maps [12]–[17]. The sequencing of the 6,132 bp unit (5,834 bp initially because an Alu sequence was missing in the original Genbank submission) failed to reveal any other coding sequence but showed a high content of interspersed repeats (comprising 62.87% of the 6.1-kb unit), including notably five Alu and one LTR (Long Terminal Repeat) sequences, this latter suspected to be involved in the origin or maintenance of the *RNU2* array [18] (GenBank accession numbers L37793 and U57614.1). In this study, the regions flanking the *RNU2* locus were also cloned and sequenced, which subsequently allowed the establishment of the gene order on chromosome 17: *BRCA1* – left junction – *RNU2* locus – right junction – chromosome 17 telomere [19].

Field Inversion Gel Electrophoresis (FIGE) analysis of >80 chromosomes from diverse human populations showed that the length of individual *RNU2* tandem arrays varied from ∼40 to ∼200 kb (∼6 to >30 repeats): 57% of them were between 100 and 200 kb (16–30 repeats), 32% were between 40 and 100 kb (6–16 repeats) and 11% were longer than the 200 kb limit of the FIGE conditions used (>30 repeats) [20]. More recently, the study of 210 HapMap individuals with Pulse Field Gel Electrophoresis (PFGE) technique revealed a wider range of allelic size (6 to more than 60 copies) [21].

The first attempts to characterise the *RNU2* array made in the eighties and the nineties were halted before CNVs started to focus the attention of scientists, certainly because its absence from the human genome reference sequence made it disappear into the dustbin of obsolete and discredited sequences. Therefore, this macrosatellite did not benefit at all from the huge acceleration in human knowledge acquisition of the last 15 years resulting from the implementation of new technologies.

Here we present the precise localization of the *RNU2* locus within the chromosome 17 reference assembly and the first direct visualisation of this highly polymorphic CNV by FISH on combed DNA.

## Materials and Methods

### Ethics Statement

The studied subjects belonged to *BRCA1* families and either carried the *BRCA1* mutation present in the family or were non-carriers [22]. Informed consent was not required as the data were analyzed anonymously. Nevertheless, the subjects belong to a study which has been reviewed and approved by the appropriate ethics committee (Comité de Protection des Personnes Ile de France III, 3 october 2006, agreement n°2373).

### Public Access Database Interrogation and Analysis of the Human Chromosome 17 Reference Sequence and of Clones' Sequence

The human genome reference sequence, working draft assemblies, clone sequences and annotations were obtained from the “UCSC Genome Bioinformatics Site” (http://genome.ucsc.edu). Gene-specific information was obtained from the “Entrez Gene” NCBI's database (http://www.ncbi.nlm.nih.gov/gene). Clone alignments were performed using the BLAST2Seq at the NCBI website (http://blast.ncbi.nlm.nih.gov/Blast.cgi?PAGE_TYPE=BlastSearch&PROG_DEF=blastn&BLAST_PROG_DEF=megaBlast&SHOW_DEFAULTS=on&BLAST_SPEC=blast2seq&LINK_LOC=align2seq).

### Cell Lines

Human lymphoblastoid cell lines (LCLs) established by Epstein-Barr virus immortalization of subject's blood lymphocytes were maintained in RPMI 1640 medium (Life Technologies, Saint Aubin, France) supplemented with 10% fetal calf serum (VWR, Fontenay sous Bois, France) and 1% penicillin– streptomycin (Life Technologies).

### Plug Preparation and Molecular Combing of DNA

EBV-immortalized lymphoblastoid cells were embedded in agarose blocks (1.2% NuSieve GTG Agarose, Lonza, Levallois-Perret, France) as previously described [23]. DNA was purified in an ESP solution: EDTA 0.5 M pH 8.0, 1% Sarcosyl (Sigma-Aldrich, Saint Quentin Fallavier, France), 2 mg/mL Proteinase K (Eurobio, Courtaboeuf, France) overnight and then agarose was melted at 68°C for 20 min and digested by 1.5 U of β-agarase (New England Biolabs, Evry, France) overnight in a M.E.S solution (2-N-Morpholino-Ethane sulfonique 500 mM pH 5.5). The resulting DNA solution was incubated with a silanized coverslip (CombiCoverslips, Genomic Vision, Paris, France), which was then removed from the solution at a constant speed of 300 µm/sec with the molecular combing system (MCS, Genomic Vision). This protocol allows maintenance of a constant DNA stretching factor of 2 kb/ µm [24]. Combicoverslips with combed DNA were then baked for 4 hours at 60°C. The quality of combing (linearity and density of DNA molecules) was estimated under an epi-fluorescence microscope equipped with an FITC filter set and a 40× air objective on freshly combed coverslips mounted in 20 µL of a 1 ml ProLong-gold solution containing 1 µL of Yoyo-1 solution (both from Life Technologies).

### Metaphase Chromosome Spreading

Metaphase spreads were prepared from patient derived lymphocytes using standard procedures.

### Probe Preparation

Probes were obtained by labelling PCR-amplified fragments using primers designed with the Primer3 v.0.4.0 software (http://frodo.wi.mit.edu/primer3/) and synthesized by Eurofins MWG Operon (Ebersberg, Germany). The entire *RNU2* repeat unit was amplified with primers ReRNU2_F/R_ (5′-GCCAAAAGGACGAGAAGAGA-3′ (59°C)/5′-GGAGCTTGCTCTGTCCACTC-3′ (60°C)) for metaphase chromosome FISH experiments. For combed DNA FISH experiments, 2 regions of the repeat unit were chosen and amplified with primers L4_F/R_ and L5_F/R_ in order to include no more than 300 bp of repeat sequences (such as Alu or LTR sequences) according to the Repeat Masker software (http://www.repeatmasker.org/cgi-bin/WEBRepeatMasker) and 4 regions flanking the *RNU2* array with primers FP1_F/R_, FP2_F/R_, FP3_F/R_ and FP4_F/R_. Long-range PCRs were performed in 20 µL reactions using Long PCR Enzyme Mix (Thermo Fisher Scientific, Illkirch, France), following these cycling conditions: 94°C for 2 min, 10 cycles of (96°C for 20 s, Tm°C for 30 s, 68°C for 45 s/kb), 25 cycles of (96°C for 20s, Tm°C for 30s, 68°C for 45 s/kb+10 s/cycle), 68°C for 10 min. Primer sequences and temperature of annealing (in brackets) were the following: L4_F_ 5′-GCGGCCCACAAGATAAGATA-3′ (59°C); L4_R_ 5′-ACGACGCAGTTAGGAGGCTA-3′ (59°C); L5_F_ 5′-CTACACAGCCCAGGACACG-3′ (59°C); L5_R_ 5′-GTTGGCCATGCCTTAAAGTG-3′ (59°C); FP1_F_ 5′-CCAAATTTTCCAAGAGACTGACTT-3′ (59°C); FP1_R_ 5′-GGAGTGAACAGGTGAGAGGATTAT-3′ (59°C); FP2_F_ 5′-GAGCCAAAAATGGATACCTAGAGA-3′ (59°C); FP2_R_ 5′-TGATCCCTGATATCCAATAACCTT-3′ (59°C); FP3_F_ 5′-TACCCCCTTCCTAGCCCTTA-3′ (59°C); FP3_R_ 5′-TCATGCAGCCTGGTACAGAG -3′ (58°C); FP4_F_ 5′-ACCGGGCTGTGTAGAAATTG-3′ (58°C); FP4_R_ 5′-ACCTCATCCTGGCTTACAGG-3′ (58°C). The sizes of the PCR fragments were 434 bp for L4, 1,959 bp for L5, 4,393 bp for FP1, 4,860 bp for FP2, 7,009 bp for FP3 and 5,340 bp for FP4. PCR products have been cloned within the pCR2.1-TOPO XL vector (Life Technologies) according to the manufacturer's instructions.

The probe used in metaphase chromosome FISH experiments was labelled with fluorescein using the nick translation method. Probes used in combed DNA FISH experiments were labelled by random-priming: 200 ng of each probe were incubated during 10 min at 100°C with 1× random primers (Life Technologies), and then cooled at 4°C during 5 min. Klenow enzyme (40U) and dNTP 1× were added. Depending on the emission color chosen, dNTPs 1mM coupled with biotin (for red emission), digoxygenin (for blue emission), or Alexa-488 (for green emission) were also added. These mixes were incubated overnight at 37°C, and the priming reaction were then stopped with EDTA 2.10^−2^ mM pH 8.

### Fluorescent In Situ Hybridization

#### On metaphase chromosomes

Hybridization was performed as described previously [25] with the probe described above and a 17q subtelomeric probe labelled with rhodamine (Cytocell, Cambridge, UK). After denaturation, overnight hybridization and post-hybridization washes, slides were DAPI counterstained and were read using a fluorescent microscope equipped with a CCD camera.

#### On combed DNA

One tenth of each random priming mix was precipitated during 1 hour at −80°C with 10 µg of Human Cot1 DNA, 2 µg herring sperm DNA, one tenth of volume of NaAc 3 M pH 5.2 and 2.5 volumes of Ethanol 100%. After centrifugation during 30 min at 4°C and at 13,500 rpm, the supernatant was discarded and the pellet dried at 37°C and dissolved with hybridization buffer (deionized formamide, SSC (salt sodium citrate) 2X, Sarcosyl 0.5%, NaCl 10 mM, SDS 0.5%, Blocking Aid). 20 µL of the mixes were laid on a coverslip with combed DNA, denatured at 95°C during 5 min, and incubation was then performed overnight at 37°C in a hybridizer (Dako, Les Ulis, France). For probe detection, hybridized coverslips were washed three times (3 min each) with formamide-SSC 2X, and three times with SSC 2X. Coverslips were then incubated 20 min at 37°C in a wet room with the first reagents: Streptavidine-A594 for Biotin-dNTP (1), Rabbit anti-A488 antibody for Alexa-A488-dNTP (2), and Mouse anti-Dig AMCA antibody for Digoxygenin-dNTP (3). Coverslips were washed with three successive baths of SSC 2X-Tween20 1%. Similarly, coverslips were incubated with the second reagents: Goat anti-streptavidine biotinylated antibody (1), Goat anti-rabbit A488 antibody (2) and Rat anti-mouse AMCA antibody (3). Coverslips were washed and incubated with the third reagents: Streptavidine A594 (1), and goat anti-rat A350 antibody (3). Coverslips were dehydrated with three successive baths of ethanol (70-90-100%). Image acquisition was performed with a customized automated fluorescence microscope (Image Xpress Micro, Molecular Devices, Sunnyvale, CA, USA) at 40× magnification, and image analysis and signal measurement were performed with ImageJ (available from NIH) and GVlab (Genomic Vision) softwares. Allelic number of copies was determined by counting the number of signals corresponding to a repeat unit only on fibres for which intact flanking probes could be observed. In all cases, the number of copies has been determined by at least two individuals, resulting in differences of one copy at the most. For the nicest 72 fibres obtained from 21 individuals, we determined the individual exact stretching factor by measuring the length of a motif covering 128 kb within the *BRCA1* bar code, which in turn allowed us to determine the physical distance separating *BRCA1* and the *RNU2* locus.

## Results

### Precise Localisation of the *RNU2* Array

**Figure 1:**
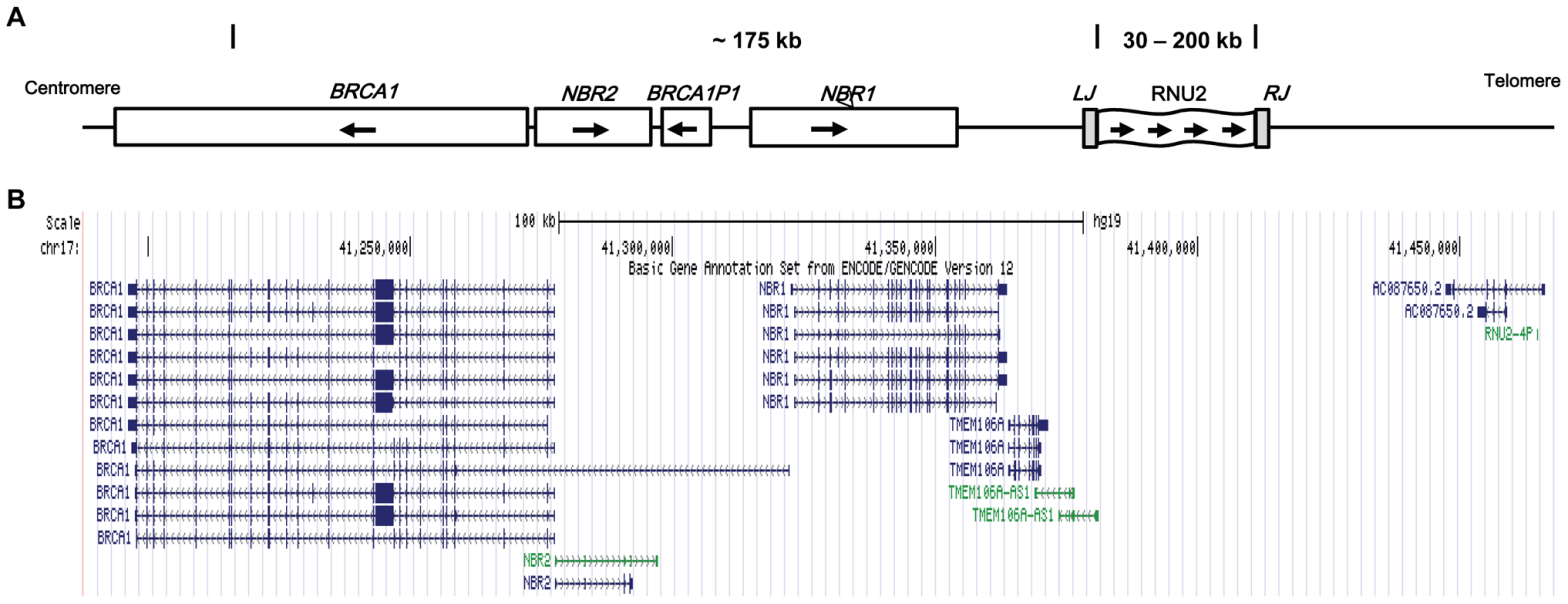
Schematic representation of the chromosome 17q21 region around the *BRCA1* gene. (A) Gene locations and physical map distances as reported in the literature [16], [19]. (B) Gene locations within a 300 Kb window as shown in the UCSC Genome Browser. Arrows indicate transcription direction. BRCA1P1: *BRCA1* pseudogene.

The organization of the *RNU2*-*BRCA1* region as published in the literature is presented in Figure 1A: the genes described within this interval are *NBR1*, *BRCA1P1* (a *BRCA1* pseudogene*)* and *NBR2* [16], [19]. The distance between the *RNU2* locus and D17S1322, a microsatellite located within *BRCA1* intron 19, is reported to be ∼175 kb based on physical maps. This would locate *BRCA1* ∼113 kb away from the *RNU2* locus. In contradiction with the literature, a single *RNU2* gene described as a pseudogene, *RNU2-4P* (289 bp long), also known as *RNU2P2*, is found on the chromosome 17 reference assembly Build 37 in the first intron of an uncharacterised gene named *LOC100130581*, ∼187 kb away from *BRCA1* (Figure 1B and Table S1 in Additional file). Along with *NBR1*, *BRCA1P1* and *NBR2,* one more gene, *TMEM106A*, has been identified by sequence analysis within this region.

**Figure 2:**
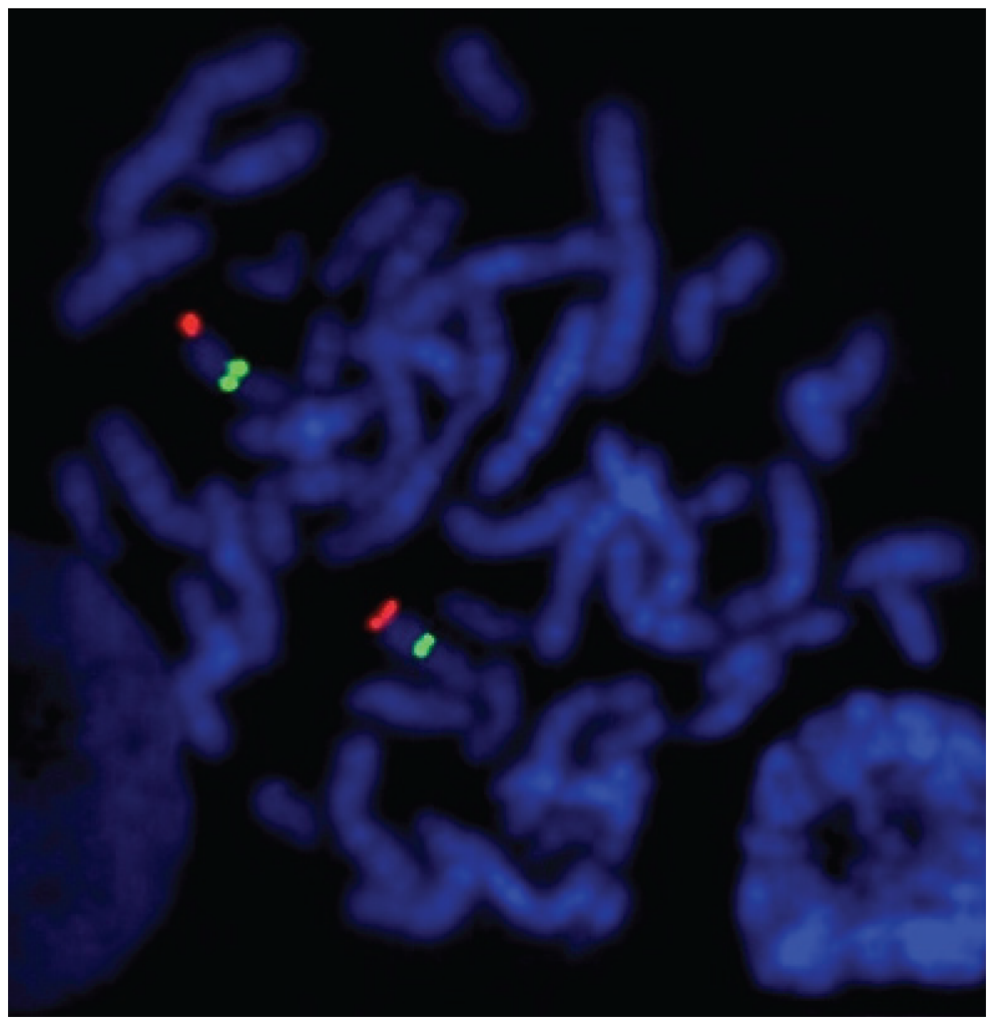
Visualization by FISH on mitotic metaphase chromosome of the *RNU2* locus. Two probes were used, one consisting of the 6.1

As shown previously [11], [26]–[27], FISH on mitotic metaphase chromosomes using a probe obtained by labelling a 6.1 kb PCR fragment amplified with primers flanking the *RNU2* repeat unit gave a unique signal over band 17q21 (Figure 2), which indicated that the repeat unit is located at the same cytogenetic band as the *BRCA1* gene. Furthermore, the high intensity of the signal was consistent with the repeat unit being present in multiple copies.

**Figure 3:**
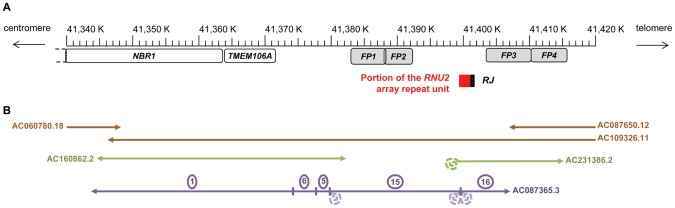
Localisation of the *RNU2* macrosatellite within the chromosome 17 sequence assemblies from NCBI Build 37.p10. (A) Schema of the region surrounding the *RNU2* array. The location of the portion of the *RNU2* repeat unit (not comprising the *RNU2* gene) and of the right junction found in the assemblies are depicted, as well as the probes used in molecular combing experiments that flank the *RNU2* array (FP1-4), and the *NBR1* and *TMEM106A* genes. (B) Clones covering the region. The reference sequence assemblies is based upon the complete sequence of 3 overlapping BACs, RP11-242D8, CTD-3014M21 and RP11-100E5 (AC060780.18, AC109326.11 and AC087650.12 respectively), represented by brown arrows. The complete sequence of the WI2-3095P13 fosmid (AC160862.2, green arrow) matches the reference sequence. The sequence of the ABC10-44487500M2 fosmid (AC231386.2, green arrow) matches the reference sequence up to its centromeric extremity where it contains several *RNU2* repeat units (depicted in a dotted curl). The five unassembled contigs of the working draft sequence of the RP11-570A16 BAC clone (AC087365.3) showing homology with the reference sequence are represented by a purple arrow. Contig 15 has been mis-assembled, as it contains several *RNU2* repeat units (depicted in dotted curls) at both its extremities.

Seven *RNU2* genes could be found in Entrez Gene (NCBI's repository for gene-specific information), among which five are considered to be pseudogenes. *RNU2-1* (GenBank accession number NR_002716.3), assigned to chromosome band 17q12-q21, is identical to the gene found in the *RNU2* repeat unit, but this locus, which in Build 36 was annotated on an unplaced contig based on a single unfinished BAC (Bacterial Artificial Chromosome) sequence, is no longer present in Build 37 as the BAC was removed from the assembly. A portion of the *RNU2* repeat unit (corresponding to positions 1440-3036 of U57614.1) is nevertheless present at position 41,399,577-41,401,198 (Figure 3A). The right junction of the *RNU2* array sequenced in 1995 [18] (416 bp: 36 bp of the repeat unit+380 bp of flanking sequence) and located telomeric to the *RNU2* locus [19] could be found as well at position 41,401,163-41,401,579, while the left junction (92 bp: 47 bp of the repeat unit+45 bp of flanking sequence) is missing from the human genome assembly, probably due to sequence assembly errors. In light of these data, we hypothesised that the *RNU2* array was located between positions 41,399,577, and 41,401,198, where the right junction and part of the *RNU2* array repeat unit can be found.

In order to sustain this hypothesis, we extracted from the databases the sequences covering this region and analyzed them. The complete sequence of a 41 kb fosmid (ABC10-44487500M2) reported in AC231386.2 confirmed the localisation of the *RNU2* macrosatellite, as it displayed 5 complete repeat units followed by sequences matching Build 37 from position 41,399,577 to 41,413,658 (Figure 3B). We also analysed the unfinished sequence of the RP11-570A16 BAC clone (AC087365.3), namely 16 unordered contigs covering 104,495 bp. Part or the entire sequence of the *RNU2* array repeat unit is found in all but contigs 1, 5 and 6. Contig 1, which contains *TMEM106A* and the end of *NBR1*, and contigs 6 and 5 match adjacent sequences on chromosome 17 (Figure 3). The main parts of contigs 15 and 16 also match adjacent sequences, with an overlap of 1.3 kb between contigs 15 and 16 corresponding to a portion of the *RNU2* array repeat unit, and of ∼500 bp between contigs 5 and 15. This ∼500 bp overlap precedes a portion of the *RNU2* array repeat unit sequence in contig 15, while it is at the end of contig 5, which suggests that contig 15 has been incorrectly assembled and that all the sequences matching the *RNU2* array repeat unit should be placed at the other end of the contig. Indeed, the assembly of these contigs is comforted not only by the chromosome 17 reference sequence assemblies but also by the complete sequence of a fosmid (AC160862.2) that covers this region. In conclusion, these data are all in agreement with a localisation of the *RNU2* macrosatellite between positions chr17∶41,399,577, and chr17∶41,401,198, which puts it ∼124 kb telomeric to the *BRCA1* gene and ∼63 kb centromeric to the *RNU2-4P* gene.

### Variation of the Number of *RNU2* Array Repeat Unit in the Human Population

**Figure 4:**
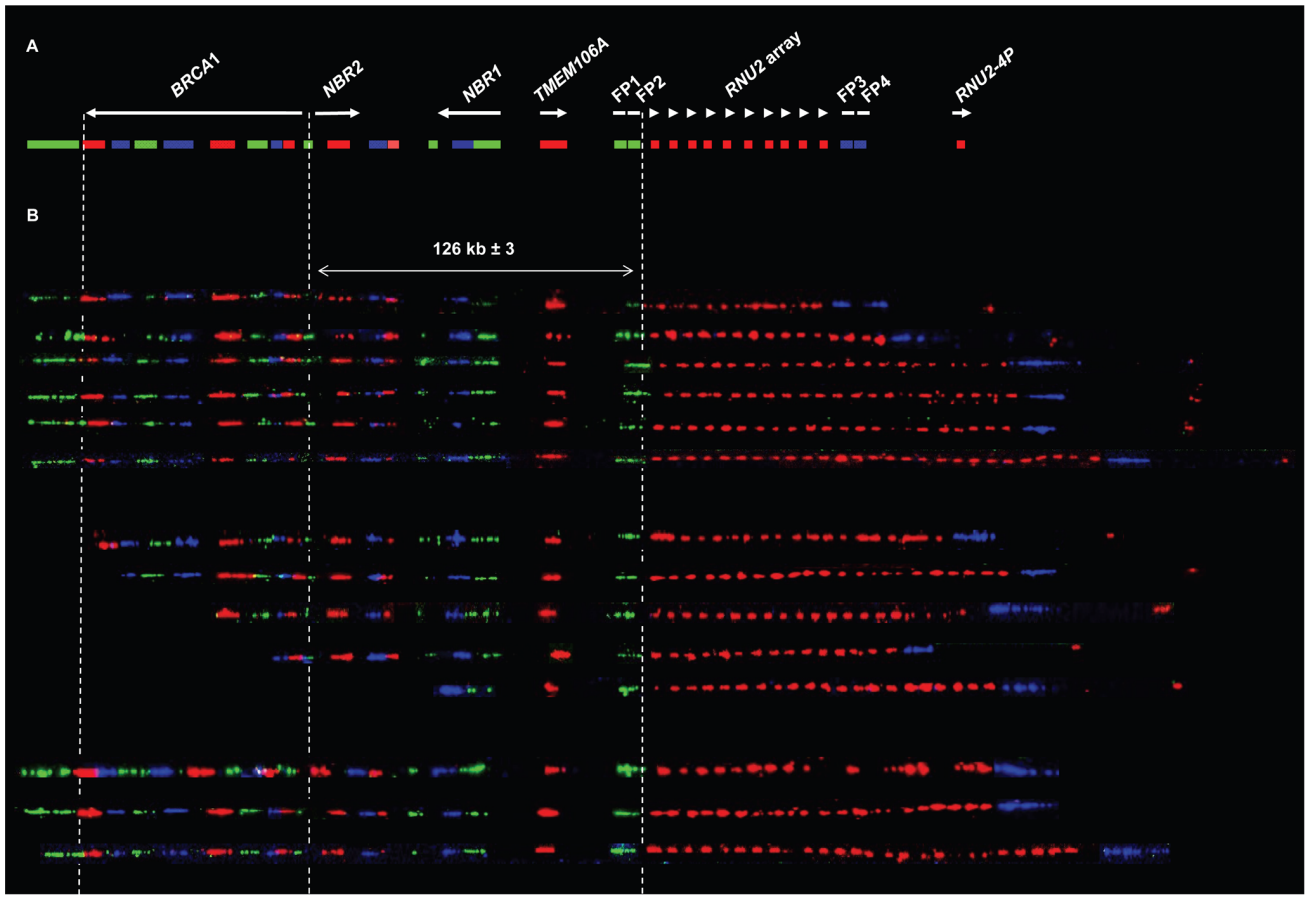
Visualization by molecular combing of the 17q21 region around *BRCA1*. (A) Schematization of the genomic morse code used. The *BRCA1* Genomic Morse Code (GMC) depicted (v4.0) is an improvement of the published code (v1.0) [23]. It covers a genomic region of 200 kb and consist in 17 signals of a distinct color (green, red or blue), each composed of 1 to 3 small horizontal bars corresponding to a single DNA probe. The signals for the flanking probes FP1-4 are each composed of 2 green or blue horizontal bars, while the signal for the *RNU2* array repeat unit is composed of 1 red horizontal bar. Of note, the probe for the *RNU2* array cross-reacts with *RNU2-4P*. (B) Fourteen fibres displaying different numbers of *RNU2* signals are shown. The first six fibres display the entire bar code from the *BRCA1* GMC to *RNU2-4P*, while the followings miss either the beginning of the *BRCA1* GMC or *RNU2-4P*.

We next undertook to directly visualize the proximity of the *RNU2* array with the *BRCA1* gene by using the molecular combing technology. We completed the existing bar code that allows to get a panoramic view of *BRCA1* and its flanking genes, namely *TMEM106A, NBR1*, *BRCA1P1*, and *NBR2* [23], with a probe obtained by labeling two PCR fragments amplified with primers flanking close regions devoid of repeat sequences within the *RNU2* array repeat unit (1.96 and 0.46 kb). We also generated four probes expected to hybridize regions flanking the *RNU2* macrosatellite based on our assumption of its location, respectively 7.3 kb downstream in the case of probes FP1 and FP2, on the centromeric side, and 2 kb upstream in the case of probes FP3 and FP4, on the telomeric side (Figure 4). Hybridisation of these probes with combed DNA of very good quality generated a consistent pattern of signals covering a genomic region >350 kb. This pattern shows the juxtaposition, from chromosome 17 centromere to telomere, of the *BRCA1* bar code, FP1-2 probes, *RNU2,* FP3-4 probes and *RNU2-4P* (the *RNU2* probes cross-react with the *RNU2* pseudogene), thus validating our tentative map (Figure 4). The average size of the interval between the end of the *BRCA1* gene and the *RNU2* array boundary was 126 kb±3 when measuring 72 fibres from 21 individuals (expected size based on the chromosome 17 reference assembly: 123.7 kb). The distance between the array boundary and *RNU2-4P* seemed consistent with that expected from our tentative map (63.4 kb), but the paucity of the number of fibres displaying both *BRCA1* and *RNU2-4P* precluded us from doing precise measures. Measurement of the *RNU2* signals gave an average size of 2.15 kb±0.63, while the average size for the gap between two *RNU2* signals was 4.30 kb±2.21, as expected on the basis of the sequence of the *RNU2* array repeat unit.

In total, we analysed 41 individuals with this technique. All but one of them displayed two populations of fibres containing different numbers of *RNU2* signals, confirming the high level of heterozygosity of the *RNU2* macrosatellite, which reached 0.98 in our small sample. Examples of fibres displaying different numbers of repeats are shown in Figure 4. The 28 different alleles that we identified among the 46 unrelated chromosomes analysed (five of which carrying a *BRCA1* mutation) are presented in Table 1: 14 of them (50%) were found only once while twelve were found twice (43%), one three times (3.5%) and one six times (3.5%). The number of *RNU2* array repeat units was found to range from 6 to 82 copies, and most of the alleles differed from their closest allele by one copy.

**Table 1:** Description of the *RNU2* array alleles identified in 46 unrelated chromosomes.

Alleles (N = 28)	Number of repeat units	Number of occurrence	Frequency of each allele
1	6	1	0.02
2	8	1	0.02
3	9	1	0.02
4	11	2	0.04
5	12	1	0.02
6	13	1	0.02
7	14	2	0.04
8	15	1	0.02
9	16	1	0.02
10	17	1	0.02
11	18	3	0.07
12	19	5	0.11
13	20	1	0.02
14	21	2	0.04
15	22	2	0.04
16	23	1	0.02
17	25	1	0.02
18	27	2	0.04
19	28	2	0.04
20	29	2	0.04
21	30	1	0.02
22	32	2	0.04
23	34	2	0.04
24	35	2	0.04
25	36	1	0.02
26	37	2	0.04
27	47	2	0.04
28	82	1	0.02

## Discussion

The gaps in the finished human genome-assemblies are likely to host undiscovered CNVs. Some long-published and well documented structural variations are also missing from human genome-assemblies due to the difficulty to assemble repeated regions [28]–[29]. Indeed repeats confuse the assembly process, often resulting in contig mis-assembly [30]. The determination of which segments of the genome are affected by CNVs and the mapping of each CNV to a human genomic region is, however, an important step to assess the phenotypic and pathologic potency of these structural variations. To date, less than a dozen macrosatellites have been characterized although this type of CNVs consisting typically of dozens of repetitive units of several kilobases are among the most polymorphic structural variations and the most likely to impact chromatin organisation and human health [31].

Here, we have determined the exact localisation of the human *RNU2* macrosatellite within chromosome 17 genome-assembly (Build 37), between positions chr17∶41,399,577, and chr17∶41,401,198, ∼124 kb telomeric to the *BRCA1* gene and ∼63 kb centromeric to one of the numerous *RNU2* pseudogenes, *RNU2-4P*, the only one present on chromosome 17. We validated this location by a FISH analysis of combed DNA (“molecular combing”) using a *BRCA1* Genomic Morse Code [23] completed by probes complementary to the *RNU2* array repeat unit and to flanking regions. This approach allowed us to determine the exact number of repeats carried by 46 independent chromosomes (41 individuals analysed in total), revealing 28 different alleles that display from 6 to 82 monomers. Up to now, two studies on the *RNU2* macrosatellite alleles have been published in which the *RNU2* array sizes were estimated from FIGE- or PFGE-separated EcoRI (a null cutter) genomic fragments visualised with a *RNU2*-specific probe [20]–[21]. Interestingly, the minimal number of repeats is the same in the three studies (i.e. 6). Liao et al. (1999) identified 15 different alleles in 28 chromosomes, but FIGE could not resolve alleles with array length >200 kb (33 copies) [20]. PFGE resolution appeared better as Schaap et al. (2013) were able to identify 58 different alleles (6–63 copies) differing from their closest allele by one copy by analysing 210 human DNA samples from four populations [21]. However, the electrophoresis-based methods may lack precision in determining the exact number of repeats for large arrays, especially for those exceeding 500 kb, while repeat number counting following molecular combing is not sensitive to array length as long as probes complementary to the *RNU2* locus flanking regions are used to assess fibre integrity. Moreover, the molecular combing technique allows the identification of possible complex repeat patterns resulting from large insertions of foreign DNA into the array and/or repeat inversions. However, electrophoresis-based methods are better suited to detect mosaicism, which is quite common in the case of macrosatellites [31]. These two techniques are therefore complementary for the study of macrosatellite repeats.

U2 snRNAs play an essential role in formation of the catalytically active spliceosome by base pairing with both the intron branch point and the U6 snRNA [32]. A five nucleotide deletion in one of the five murine U2 snRNA genes causes ataxia and neurodegeneration, neuron loss being strongly dependent on the dosage of wild-type and mutant U2 snRNAs [33]. This finding suggests that *RNU2* might be associated with disease in humans as well. Growing evidence links splicing factor dysfunction with disease, particularly cancer [34]. Furthermore, the proximity of this macrosatellite to the *BRCA1* gene combined with its high degree of polymorphism raise the interesting possibility that it could be involved in breast cancer susceptibility. Indeed, investigations in mice have suggested that the effect of CNVs on the expression of flanking genes could extend up to 450 kb away from their location, all the more in the case of long CNVs (> 50kb) [35]. In humans, the stronger evidence of such an effect so far came from the study of the Williams-Beuren syndrome, where not only hemizygous genes that map within the microdeletion responsible for the disease but also normal copy neighboring genes show decreased relative levels of expression [36]. Our study, which gives a precise localization and better characterizes the *RNU2* locus, provides the foundation for testing the association between copy number at this locus and breast cancer or other diseases risk.

## Supporting Information

Table S1 **Genomic coordinates of 17q21 genes and sequences (Build 37.p10).**
(DOCX)
