# Supplementary material for: Direct Visualization of the Highly Polymorphic RNU2 Locus in Proximity to the BRCA1 Gene
Source: PLoS One. 2013 Oct 11;8(10):e76054. doi: 10.1371/journal.pone.0076054 (PMC3795722; doi:10.1371/journal.pone.0076054)
Supplement: Table S1 — Genomic coordinates of 17q21 genes and sequences (Build 37.p10). (DOCX) [file pone.0076054.s001.docx]

**Table S1.** Genomic coordinates of 17q21 genes and sequences (Build 37.p10).

| **Gene or sequence name** | **Genomic coordinates** |
| --- | --- |
| *BRCA1* | Chr17:41,196,312 – 41,277,500 |
| *NBR2* | Chr17: 41,277,600 – 41,292,342 |
| *NBR1* | Chr17: 41,323,246 – 41,363,707 |
| *TMEM106A* | Chr17: 41,363,894 – 41,371,589 |
| FP1 | Chr17:41,383,743 – 41,388,135 |
| FP2 | Chr17:41,387,378 – 41,392,237 |
| FP3 | Chr17:41,403,257 – 41,410,265 |
| FP4 | Chr17: 41,410,222 – 41,415,561 |
| *LOC10030581* | Chr17: 41,447,213 – 41,466,266 |
| *RNU2-4P* | Chr17:41,464,594 – 41,464,785 |
| *ARL4D* | Chr17: 41,476,353 – 41,478,504 |
